# Advanced Hydrogels for the Controlled Delivery of Insulin

**DOI:** 10.3390/pharmaceutics13122113

**Published:** 2021-12-08

**Authors:** Shazia Mansoor, Pierre P. D. Kondiah, Yahya E. Choonara

**Affiliations:** Wits Advanced Drug Delivery Platform Research Unit, Department of Pharmacy and Pharmacology, School of Therapeutic Sciences, Faculty of Health Sciences, University of the Witwatersrand, 7 York Road, Parktown, Johannesburg 2193, South Africa; 707825@students.wits.ac.za (S.M.); pierre.kondiah@wits.ac.za (P.P.D.K.)

**Keywords:** hydrogels, injectable hydrogels, stimuli responsive systems, controlled release, insulin administration

## Abstract

Insulin is a peptide hormone that is key to regulating physiological glucose levels. Its molecular size and susceptibility to conformational change under physiological pH make it challenging to orally administer insulin in diabetes. The most effective route for insulin delivery remains daily injection. Unfortunately, this results in poor patient compliance and increasing the risk of micro- and macro-vascular complications and thus rising morbidity and mortality rates in diabetics. The use of 3D hydrogels has been used with much interest for various biomedical applications. Hydrogels can mimic the extracellular matrix (ECM) and retain large quantities of water with tunable properties, which renders them suitable for administering a wide range of sensitive therapeutics. Several studies have demonstrated the fixation of insulin within the structural mesh of hydrogels as a bio-scaffold for the controlled delivery of insulin. This review provides a concise incursion into recent developments for the safe and effective controlled delivery of insulin using advanced hydrogel platforms with a special focus on sustained release injectable formulations. Various hydrogel platforms in terms of their methods of synthesis, properties, and unique features such as stimuli responsiveness for the treatment of Type 1 diabetes mellitus are critically appraised. Key criteria for classifying hydrogels are also outlined together with future trends in the field.

## 1. Introduction

β-cells located within the pancreatic islets of Langerhans produce insulin (a peptide hormone) that regulates physiological glucose homeostasis. However, in diabetic patients, hormone functioning is impaired, resulting in uncontrolled hyperglycemia [1]. The autoimmune destruction of pancreatic β-cells leads to type 1 diabetes (T1D), in which there is a total lack of insulin secretion. Exogenous insulin remains the most effective form of treatment for the management of T1D, often requiring multiple daily injections. This often results in poor patient compliance, resulting in increasing mortality and hospitalization rates [2].

To overcome the barriers facing insulin administration, insulin analogues and innovative delivery systems have been developed to manipulate the pharmacokinetic profile of various insulin analogues. Orally administered insulin is desirable, but the poor GIT stability and large molecular weight (size) limit the possibility of oral administration of insulin to reach sufficient bioavailability [3]. Thus, injectable insulin remains the gold standard for T1D therapy, and much research has concentrated on the design of injectable hydrogel-based systems for the controlled (sustained) release of insulin [4]. Injectable hydrogels undergo a sol–gel transition and are typically administered in the sol state using a syringe with transitioning to a gel in vivo [5]. Insulin can be encapsulated within nanoparticles (NPs) fixated in the hydrogel before injecting. Once sol–gel transitioning occurs, the insulin is entrapped within the hydrogel network to provide spatio-temporal release over time [6]. This strategy allows for fewer injections to be administered with a reduced side effects profile and an improved patient compliance.

**Figure 1:**
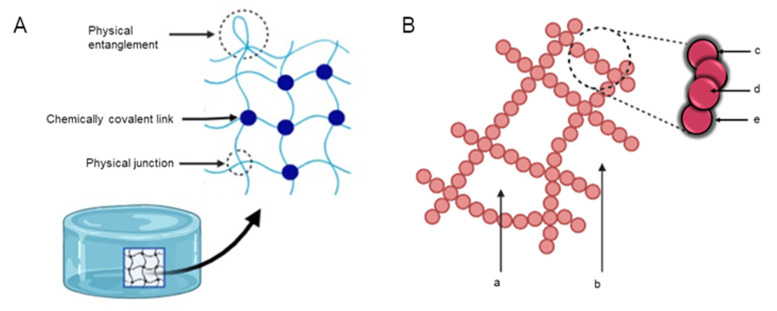
(**A**) Annotated illustration showing the mesh structure of a hydrogel. Physical and chemical points of crosslinking are illustrated along with physical entanglement of the structure. (**B**) Illustration depicting the morphology of a hydrogel network. (a) Interstitial water, (b) Free water, (c) Bound water, (d) Hydrogel monomer represented by spheres, (e) Semi-bound water. Adapted from [9,10], published by Elsevier, 2015 and Taylor and Francis, 2015.

Hydrogels are 3D polymeric matrices and take on a solid matrix structure known as the first phase (Figure 1A) [7]. When encountering fluid, the molecules penetrate and diffuse into the matrix, causing the networked chemical structure to relax and resulting in a barrier-like 3D meshed structure filled with fluid in the interstitial space (Figure 1B) [8]. The hydrogel structure can impart an elastic force that allows hydrogels to expand and contract for added structural stability. The ionic phase comprises ionizable groups that are bound to the polymer chains and allow for functionalization of the hydrogel system [9]. Biodegradable, natural, and synthetic polymers (or combinations of these) are frequently used to synthesis hydrogels. Hydrogels that can retain considerable amounts of water have hydrophilic functional groups on their polymer backbones such as –OH, –NH_2_, or –COOH [6,10]. Water retention (swelling) results in desirable consistency and interfacial tension with biological fluids [7,11]. Figure 1B depicts the molecular structure of a typical hydrogel and its interactions with water. Interestingly, the widespread use of hydrogels in biomedical applications is largely due to their morphology being similar to the extracellular matrix (ECM) of the body [7,8].

Hydrogels do not impact metabolism and are designed to be bio-orthogonal in vivo. They are biocompatible and have a porous structure and tunable biodegradability [8]. The pore size determines the mesh size, which can be controlled to achieve the sustained delivery of bioactives [12]. Thus, the rate of bioactive delivery is dependent on the swelling and stimuli responsiveness of the hydrogel. Stimuli-responsive hydrogels can respond to triggers such as physiological temperature and pH. Injectable hydrogels, which are a specialized class of hydrogels, can be administered subcutaneously or at the target site to avoid presystemic metabolism. The nature of stimuli-responsive injectable hydrogels to transition into a viscoelastic gel once injected can serve as a depot (reservoir) for controlled insulin release [6].

**Figure 2:**
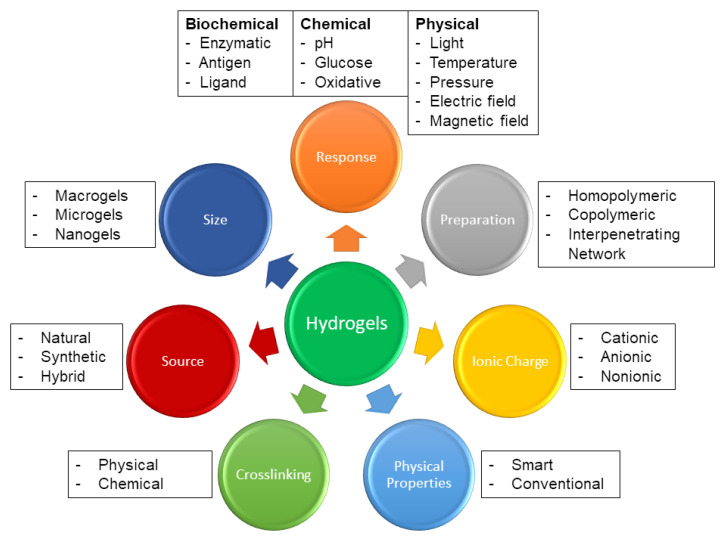
An illustration of how hydrogels can be classified based on factors such as the source and preparation of polymers employed, the charge of hydrogels, type of crosslinking, stimuli responsiveness, and physical properties. Adapted with permission from [9]; Published by Elsevier, 2015.

Hydrogels are classified widely based on the source of polymer/s employed, ionic charge of bound group/s, type of crosslinking (physical or chemical), stimuli responsiveness, synthesis methods, biodegradability, and structure (Figure 2) [9]. The utilization of hydrogel platforms for the advanced delivery of therapeutic proteins and peptides has revolutionized therapeutics and is an exponentially growing class of treatment.

Therefore, this review provides a concise incursion into recent developments for the safe and effective controlled delivery of insulin using advanced hydrogel platforms with a special focus on the sustained release injectable formulations. Various hydrogel platforms in terms of their methods of synthesis, properties, and unique features such as stimuli responsiveness for the treatment of T1D are critically appraised. Key criteria for classifying hydrogels are also outlined together with future trends in the field.

## 2. Biocompatibility and Biodegradability of Hydrogels as Insulin Carriers

### 2.1. Biocompatibility

Insulin has a quaternary structure made up of A and B chains and is preserved via weak non-covalent bonds. First-pass metabolism usually occurs when insulin is administered orally. Insulin enters the portal vein and travels via the small intestine, colon, and spleen to reach the liver, where it may be metabolized, before entering the systemic circulation. This leads to a lower drug bioavailability [13,14]. The weak covalent bonds of insulin are affected by the pH of the gastrointestinal tract (GIT) and stomach and are degraded physically and chemically. Additionally, insulin is a macromolecule resulting in a reduced drug bioavailability [15,16]. To improve the bioavailability of insulin, researchers have incorporated the drug into the mesh structure of the hydrogel system. Thus, the peptide is protected by the hydrogel network and is injected to avoid the GIT for protection from physiological destabilization and degradation. Scientists employ specific polymers that have tunable properties in hydrogel synthesis so as to circumvent a toxic or immune response in the body [16,17], thus rendering the insulin encapsulated hydrogel: a biocompatible drug delivery system (DDS).

### 2.2. Biodegradability

Formulated hydrogels are designed so that they are able to self-degrade, allowing for low or non-toxicity build-up over time [8]. This means that the hydrogel DDS synthesized can be metabolized into non-toxic by-products or expelled via glomerular filtration [18]. Hydrogels have the advantage of biodegradability in comparison to other biomaterial platforms due to the polymers employed [8]. Furthermore, labile bonds (Table 1) that have been introduced into the hydrogel system, as either the cross-linker or in the network backbone, are cleaved along with unstable linkages. This is accomplished primarily via hydrolysis or under physiological conditions, either chemically or enzymatically [18]. When a biodegradable hydrogel has been loaded with a drug, the DDS has an added advantage of sustained release over a significant period of time, improving patient compliance as well as reducing side effects and morbidity rates [19]. Overall, functional groups such as amines, esters, anhydrides, phosphazenes, and phosphate esters, which are found in the polymeric and biomolecule components, can influence hydrogel degradability [20,21].

The use of carbohydrate polymers, which are biodegradable, enables controlled hydrogel degradation while releasing insulin in a sustained manner. Hydrogels made from polysaccharides i.e., carbohydrates, have shown substantial progress in targeted insulin delivery due to their natural characteristics such as pore size, mesh structure, sustainability, and stimuli responsiveness to external factors [17].

Carbohydrate polymers may also be modified to produce synthetic polymers, such as methylcellulose or carboxymethyl cellulose (CMC), for use in hydrogel preparation, as was done by Gao et al. The team of researchers developed CMC/polyacrylic acid (PAA) hydrogels for orally administered insulin (60 IU/kg). In vivo studies carried out in diabetic rats demonstrated that fasting plasma glucose levels decreased by 72.4% within 6 h after administration. Additionally, the relative bioavailability of the orally administered insulin loaded hydrogel (6.59%) was 10× greater than of free insulin solution (>0.5%) [31].

In 2020, researchers further used carbohydrate polymers for insulin delivery. They developed a pH-sensitive carboxymethyl β-cyclodextrin grafted carboxymethyl chitosan hydrogel. Using EDC/NHS as coupling agents, the hydrogel was synthesized for oral insulin administration. Cell studies showed non-toxicity, while in vivo studies demonstrated a lowered blood glucose level (BGL) that was significant and sustained (6–12 h) [32].

The use of synthetic polymers for the oral administration of insulin shows promise. However, shortfalls such as a lack of sustained drug release and increasing drug release profiles in response to physiological pH levels remain obstacles.

## 3. Hydrogel Synthesis, Morphology, and Properties

### 3.1. Physical and Chemical Crosslinking

As hydrogels are networks or matrices of polymers, crosslinking between the polymers is required to prevent the dissolution of the network before use [18]. The type of crosslinking used in hydrogel synthesis is an important factor that affects hydrogel properties and must be determined in accordance with its desired application [6]. Polymeric crosslinking can be largely classed as either physical or chemical. When hydrogels swell, the crosslinking of polymers assists in maintaining the 3D conformation of the gel [33]. Crosslinking may occur; in vitro i.e., during hydrogel synthesis, or in vivo (in situ) i.e., post administration of the hydrogel [34]. Hydrogels possess physical domain junctions under physical crosslinking, and a non-permanent self-assembly takes place. Chemical crosslinking takes place covalently between the hydrogel polymer chains [9]. As crosslinking is introduced, the hydrogel exhibits viscoelastic or purely elastic properties. The type of crosslinking should be determined by the polymerization technique employed in the formulation as well as the desired features of the hydrogel system. In terms of toxicity occurring during chemical crosslinking, the crosslinker used in the hydrogel’s synthesis can be removed from the system before usage by dialysis or any other suitable method [18].

Dual-network hydrogels may be formed because of electrostatic interactions and are synthesized via a combination of physical and chemical crosslinking. These hydrogels have been developed to overcome the shortfalls of using only physical or chemical crosslinking. These dual-network hydrogels have a higher swelling capacity over a wider pH range and are more sensitive to pH fluctuations than chemically cross-linked hydrogels [9].

### 3.2. Morphological Components

A hydrogel’s morphology is determined primarily by the amount of water it can retain and the crosslinking process used. Depending on the concentration of hydrophilic groups occurring on the polymer chains, hydrogels may retain different quantities of H_2_O. The amount of water retained by the hydrogel varies from a fraction to a 1000× its weight [8]. The swelling ability of hydrogels is critical for their usage in biomedical applications. This is due to the equilibrium swelling ratio to the ratio of the weight of swollen hydrogel divided by the dry hydrogel, which affects multiple parameters. These parameters include the solute diffusion coefficient, mechanical properties, surface wettability, and mobility of the hydrogel. The molecular weight (MW) and charge of the polymer/s employed in hydrogel synthesis regulate the physical properties of swollen hydrogels. Moreover, the density of crosslinking and physical associations also plays a role in the physical characteristics of the swollen hydrogel. Furthermore, the amount of bonding between polymeric chains is defined by each of these parameters. The integrity of encapsulated therapeutic agents is dependent on these properties as protection from mechanical deformation at the targeted drug delivery site [35,36].

### 3.3. Molecular Characteristics

#### 3.3.1. Viscoelasticity

As polymer chains crosslink, the hydrogel network exhibits viscoelastic and/or purely elastic behavior [18]. However, before gelation of the hydrogel matrix, the viscosity must be low enough to permit a homogenous dispersion of medication within the gel. After injecting of the hydrogel system, moderate gelation conditions must exist in order to minimize cytotoxicity and sufficiently encapsulate pharmaceutical drugs and cells to be administered.

As a result, the viscosity of a polymer solution needs to be taken into account to achieve a hydrogel solution that is easily injectable, thereby allowing for a non-surgical procedure that is minimally invasive [6].

#### 3.3.2. Pore Size

The porosity of hydrogels refers to a highly intertwined and organized mesh structure that makes up the hydrogel matrix. This network may exist at the micro- or nano-scale depending on the use of the hydrogel [6]. The porosity of a hydrogel is regulated and dependent upon the crosslinking density of the matrix network. The porous morphology provides a matrix for drug loading while also protecting the drug from any physiologically damaging environments [8]. These porous matrices are important for improved drug release as well as for the transport of nutrients for cell growth if need be [6]. The porous network may vary in its distribution, size, and interconnections while making up the hydrogel structure. ‘Tortuosity’ may be used to describe these three characteristics, and this along with the hydrogel composition and amount of crosslinking affects the hydrogel tortuosity [37].

A hydrogel’s porous network can be categorized as nonporous, microporous, or superporous as related to the pore size between the polymer networks. The porosity of the gel has a significant impact on its swelling behavior. Nonporous hydrogel diffusion is the only way to achieve water swelling in a medium. Microporous hydrogels have very small pores ranging from a few microns to a few hundred microns, which can be investigated using SEM or other imaging technology. Accordingly, there is an increased diffusion rate of swelling in microporous hydrogels as water uptake takes place via diffusion and/or leaching through pores. Specifically formulated superporous hydrogels have concentrated, larger pore sizes, thereby allowing for higher swelling rates over a shorter period. The commercial applications for these types of hydrogels include superabsorbent responsive biomedical devices, agriculture, and others [10].

The pore size of the hydrogel to be synthesized must be chosen with care, taking into consideration the diffusion and swelling rates desired. The larger and more concentrated the pore size, the faster the swelling mechanism [10].

#### 3.3.3. Swelling and Drug Release

**Figure 3:**
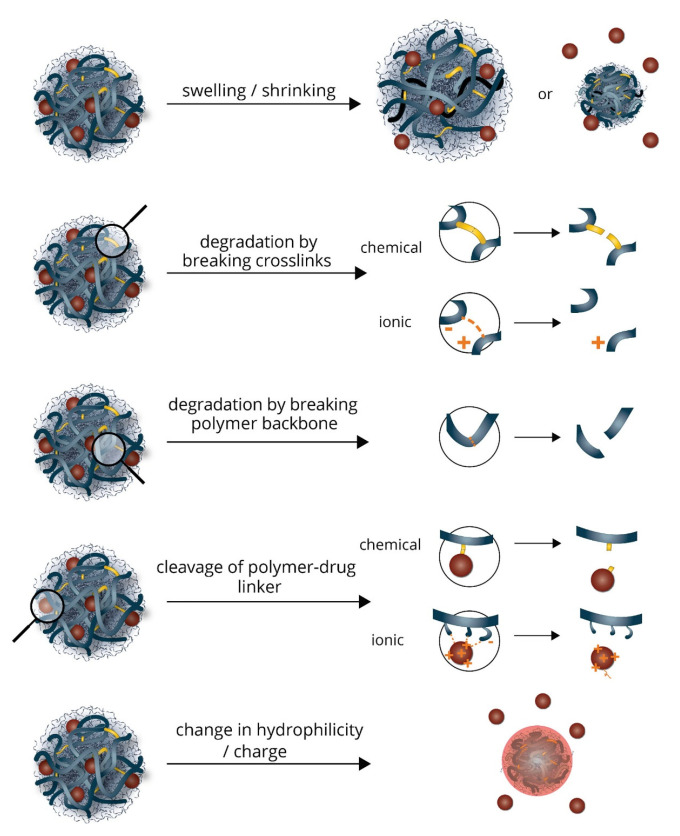
Illustration of the varied stimuli-responsive swelling mechanisms occurring within hydrogels. Adapted with permission from [39]; Published by Elsevier, 2019.

Due to the hydrophilic chains in the hydrogel network, the hydrogel has the ability to take up water and swell [11]. The uptake of water into the hydrogel can be controlled by stimuli responsiveness [3]. The ability to retain water is very important, as the hydrogel mimics human tissue and thus is considered favorably in a host of biomedical drug delivery platforms. Due to crosslinking, hydrogels are able to maintain their 3D conformation during swelling [9]. Stimuli-responsive swelling is synonymous with the control of diffusion into and out of the hydrogel network, which in turn allows for spatial and temporal control over drug release, as seen in Figure 3 below [3]. The mechanism of hydrogel swelling is based upon three differing concepts: The Differentiation Theory, The Mechanism of Gelation, and The Rubber Elasticity Theory. The Flory–Huggins Theory discusses how temperature and polymer–H_2_O interactions affect the swelling magnitude of a hydrogel. The release rate of encapsulated drugs is an important factor to determine when formulating the hydrogel. The release rate of drugs is dependent largely on the diffusion coefficient of the pharmaceutical agent through the gel structure [8,38]. Zero-order drug kinetics is the golden standard for release rates.

## 4. Modified Hydrogel Platforms for Controlled Insulin Delivery

### 4.1. Injectable Hydrogel Systems for the Regulated Delivery of Insulin

There have been numerous studies conducted on injectable hydrogels as insulin carriers for the management of T1D.

Xue developed an injectable shear-thinning hydrogel to encapsulate and modulate the release of insulin. A cyclodextrin–adamantane supramolecular hydrogel was used to induce the spontaneous crosslinking of hyaluronic acid (HA) and showed that the hydrogels formed an injectable depot in vivo. It was also shown that insulin release was controlled in vitro and in vivo by altering the molecular mass and concentration of the polymers. In addition, the hydrogels could be blended with protein-based insulin particles to extend the release rate in vivo and in vitro over 30 days [40].

Dong et al. produced a stimuli-responsive injectable hydrogel based on the complexation of glucose and boronic acid in which the ratio of boronic acid to glucose functional groups was paramount for the synthesis. Polymers with 10–60% boronic acid and the rest glucose-modified were favored. The hydrogel showed shear thinning and self-healing to rebound from a shear-induced flow to a gel state instantaneously and showed glucose-responsive in vitro release [41].

A novel glucose-responsive insulin encapsulated injectable hydrogel platform was synthesized by Zhao et al. The hybrid formulation combined phenylboronic acid-poly (lactic-co-glycolic acid) (PBA-PLGA) modified microparticles with a HA–dopamine hydrogel. The hydrogel was developed with shear-thinning behavior to allow for injection and tissue-adhesive properties and demonstrated rapid glucose responsiveness at physiological pH. When injected once subcutaneously in vivo into STZ-induced diabetic mice, blood glucose was sustained for up to 2 weeks [42].

Other research groups have developed a trehalose-based injectable hydrogel for insulin to preserve the structure of insulin when delivered and protect it from high temperature. A polymer containing trehalose side chains with a PBA end-functionalization polyethylene glycol (PEG) was employed. Boronate ester crosslinks with the PEG–boronic acid via the –OH groups on the trehalose side chain formed the hydrogel. The addition of glucose as a stronger binder to boronic acid caused stimuli-responsive insulin release. Results showed that trehalose-based polymers and boronic acid cross-linkers can be used to rapidly synthesize injectable hydrogels. The degradation rate of the hydrogel was accelerated by increased glucose content in the buffer inducing rapid insulin release. In addition, the trehalose hydrogel proved to be an excellent heat stress protector for insulin. Since protein-based therapies must be kept at specific temperatures to maintain activity, trehalose hydrogels could potentially be used without the need for specialized refrigeration. Within 1 min, the mixture thickened, and within 5 min, a gel was formed, making it suitable for injection [43].

An injectable silk fibroin hydrogel (iSFH) has also been developed by Maity et al. to provide a prolonged release of insulin. Ethylene-and triethylene-glycol was added to the silk fibroin protein, forming an injectable hydrogel within 50 min. The desired encapsulation of active insulin was demonstrated via SEM in which the hydrogel showed a mesoporous structure. T1D Wistar rats were injected subcutaneously with the insulin–iSFH system, resulting in a regulated release of insulin and maintenance of normoglycemia for up to 4 days. The biodegradable and biocompatible nature of iSFH has increased interest in using it as a potential system to regulate and prolong the delivery of insulin, thus achieving homeostatic blood glucose levels [44].

Injectable hydrogels are able to deliver targeted drug delivery with sustained release while maintaining physiological BGLs. As demonstrated above, the injectable systems are often stimuli responsive, forming a depot within the body, virtually mimicking the pancreas [6]. These injectable hydrogels also do not have the expense of 3D printing. However, for a system that is completely biomimetic, more research needs to be done with regard to incorporating islet stem cells into the hydrogel systems for β-cell regeneration [35]. In addition to injectable hydrogels, microgels and nanogels are also being studied extensively (Table 2).

### 4.2. The Use of Microgels for the Controlled Delivery of Insulin

Microgels are 3D microscopic intramolecular cross-linked gels that are macromolecules with a modifiable shape and size range of 1 nm to 10 µm [51]. Microgels are distinct in that they are injectable and offer a highly tunable, modular microstructure while retaining the bioactive encapsulating properties and biocompatibility of the hydrogel analogue. Microgels are extensively tunable and demonstrated their relevance in a wide range of tissue engineering applications [52]. They can be synthesized via minor modifications to established polymerization protocols and can be designed to have pendant functional groups capable of interacting with metal ions, which are progenitors of metal nanoclusters. Thus, microgels can be loaded with metal precursors, which then are reduced inside the microgel to yield metal particles [53]. The colloidal stability in conjunction with a Young’s modulus of 0.1–100 kPa allows for the excellent loading and release of encapsulated bioactives within the microgel network [54].

Volpatti et al. synthesized a microgel encapsulating nanoparticles for specialized glucose-responsive insulin release. Alginate was cross-linked with divalent cations to form the microgel platform, and the nanoparticles were synthesized using acetylated-dextran, glucose oxidase, and catalase before loading insulin. The alginate-based microgel demonstrated the ability to maintain glucose-responsive insulin release in preclinical testing with homeostatic blood glucose levels achieved using two doses over a period of 21 days in vivo [45].

**Figure 4:**
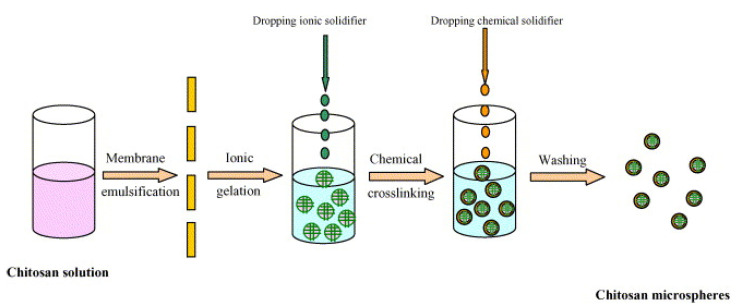
Diagram showing the synthesis of insulin-loaded CHC microspheres via ionic crosslinking to produce microgels in conjunction with chemical crosslinking. Adapted with permission from [46]; Published by Elsevier, 2006.

Researchers also developed a microgel containing insulin-loaded chitosan (CHC) microspheres (Figure 4). By transferring an aqueous CHC/acetic acid solution loaded with insulin via the uniform porous glass membrane into a paraffin/petroleum ether combination comprising hexaglycerin penta-ester (PO-500) emulsifier, a stable W/O emulsion with a consistent particle size was produced. Ionic crosslinking with tripolyphosphate (TPP) added dropwise to the emulsion generated a microgel. Chemical crosslinking with glutaraldehyde was undertaken to harden the homogenous droplets of CHC. The microspheres were solidified to form a gel by ionic crosslinking between TPP and CHC, and the state of solidification was adjusted in vitro based on encapsulation efficiency, microsphere shape, drug activity, and the insulin release profile. The final insulin-loaded microgel was evenly sized and ranged in diameter from 4 to 15 mm in diameter [46].

**Figure 5:**
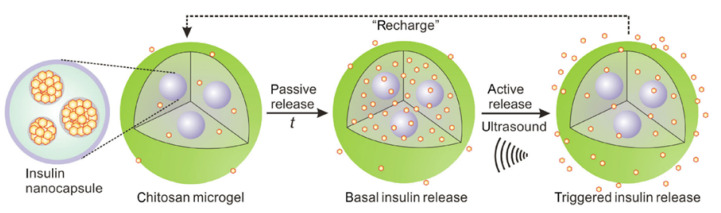
Schematic diagram of a CHC microgel encapsulating insulin-loaded PLGA nanocapsules. The loaded insulin has a passive and active basal drug release but is triggered and dependent on US. Adapted from [47] (Creative Common License); Published by Springer, 2017.

Di et al. developed insulin-encapsulated PLGA nanocapsules loaded (via passive diffusion) within CHC microgels. The ultrasound-triggered system allowed for a pulsatile release of insulin via the microgel, allowing for both a rapid acting and sustained insulin release over time. Insulin was promptly released after ultrasound treatment to control blood glucose levels (Figure 5). In in vivo studies in T1D mice (subcutaneously injected) with the insulin-loaded microgel (250–300 μg/mL per mouse) and thereafter, the application of ultrasound demonstrated the successful maintenance of blood glucose levels for 7 days when activated by 30 s of ultrasound administration [47].

### 4.3. The Use of Nanogels for the Controlled Delivery of Insulin

Similar to microgels, nanogels have a swellable 3D structure but at the nanoscale dimension (10–1000 nm). Nanogels are formed by physical or chemical crosslinking of natural or synthetic polymers and have a significant capacity to retain water without dissolving due to the presence of hydrophilic or amphiphilic macromolecular chains. Nanogels have water content that corresponds with the fluid-like transport characteristics of physiologically active compounds that are significantly lower than the gel pore size [55,56]. Stimuli-responsive nanogels specifically have great potential for application in controlling the delivery of bioactives due to their size, tuneability, and biocompatibility [39]. Nanogels are also known as hydrogel nanoparticles and act as multifunctional complexes with adaptable properties. Nanogel-based formulations are useful in nanomedicine [55].

**Figure 6:**
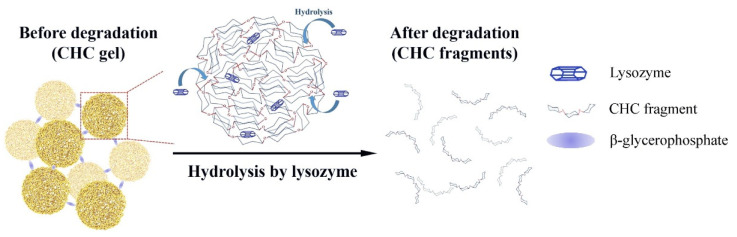
Illustration of lysozyme-dependent CHC gel degradation and insulin release. Adapted with permission from [48]; Published by Elsevier, 2016.

An insulin-loaded injectable nanogel was developed by Chou et al. using self-assembled nanoparticles comprising carboxymethyl-hexanoyl CHC. The researchers combined carboxymethyl-hexanoyl CHC nanoparticles with β-glycerophosphate, which is a natural compound found within the body. It intercalates with CHC and acts as a catalyst, allowing the system to form a matrix network that can undergo gel–sol transition under high temperatures [57]. Additionally, researchers incorporated lysozyme to the system to regulate insulin release and nanogel biodegradation (Figure 6). The presence of active lysozymes on CHC was verified by in vitro assays and showed that the quantity of lysozymes in the nanogel affected the rate of insulin release. Cytotoxicity results revealed that the nanogel was biocompatible, while animal studies showed a single injection maintained fasting blood glucose for 10 days. The insulin-loaded CHC–lysozyme nanogel showed much promise as an injectable gel for sustained insulin delivery with the ability to control basal insulin levels over a prolonged period [48].

The in vivo application of a nanogel-based insulin delivery systems was explored by Wu et al. employing poly (3-acrylamidophenyl boronic acid), poly (*N*-isopropylacrylamide), and dextran polymers in the synthesis of a nanogel. Subcutaneous injections of the MG3 insulin-loaded nanogel (4 IU kg/BW) as well as native insulin at a dose of 2.5 IU kg/BW were administered to T1D rodents. The nanogel with a self-regulating 150 nm range was responsive to high blood glucose levels, and its swelling resulted in insulin release. In vivo animal studies revealed increased efficacy in controlling blood glucose levels for 2 h [49].

Lee et al. synthesized a glucose-responsive nanogel loaded with insulin by employing sodium alginate poly (L-glutmate-co-*N*-3-L-glutamylphenylboronic acid) and glycol CHC to synthesize a double-layered nanogel. In vitro cytotoxic assays and in vivo results demonstrated that the nanogel was biocompatible and glucose responsiveness was achieved [50].

## 5. Stimuli-Responsive Hydrogels for Controlled Insulin Delivery

Hydrogels containing glucose sensing carriers that trigger insulin release are designed to respond to varying blood glucose levels [58]. Switchable sol–gel transitions are triggered by stimuli-sensitive hydrogels with properties that facilitate a response to changes in their environment [59]. External factors that cause transitions in state can be physical, chemical, or biological. Stimuli-responsive hydrogels react to small changes in environmental variables that can include light, temperature, ionic strength, pH, electrical, and magnetic fields [60,61]. Among stimuli-responsive triggers, pH and temperature responsiveness have received much attention due to their human physiological relevance and can be regulated and assayed under both in vitro and in vivo settings [35,62]. Stimuli-responsive hydrogels can also control the release of bioactives in response to specific triggers [63]. They can be synthesized by combining stimuli-responsive polymers such as alginate (enzymatic, ionic concentration), CHC (pH, enzymatic), poly(*N*-isopropylacrylamide), pluronics (temperature), and others. Pluronic or poloxamer is a synthetic polymer that takes the form of a gel at high temperatures. Due to hydrophobic interactions, pluronic is a liquid while at cold temperatures. Due to the hydroxyl functional groups at the ends of the chain, chemical changes may easily take place. This synthetic polymer demonstrates gel integrity with extended incubation. This is advantageous for the long-term delivery of drugs, as it allows for sustained drug release [64]. These responsive polymers react rapidly to stimuli, resulting in structural modifications such as form or characteristics, solubility, and sol–gel transitioning in a reversible or irreversible manner [5]. Glucose-sensitive hydrogels have also been studied based on their desirable prospects for developing insulin delivery systems that can function as an artificial pancreas and delivering a precise quantity of insulin in response to blood glucose concentrations [35].

### 5.1. Glucose Oxidase Stimuli Release Systems

Glucose-responsive systems release bioactives proportionally to blood glucose concentration. When glucose oxidase (GOx) is coupled with pH-responsive polymers such as CHC, the enzymatic oxidation of glucose to gluconic acid catalyzed by GOx in the glucose solution causes the pH of the microenvironment to alter. Then, the pH shift causes the GOx-incorporated hydrogel to expand or shrink. When glucose is changed to gluconic acid by GOx while in the presence of O_2_, the local pH of the system drops, increasing the swelling of cationic hydrogels and releasing insulin. GOx has been covalently bonded onto a hydrogel to decrease the burst release of insulin in the system and promote the regulated loading of insulin [9].

**Figure 7:**
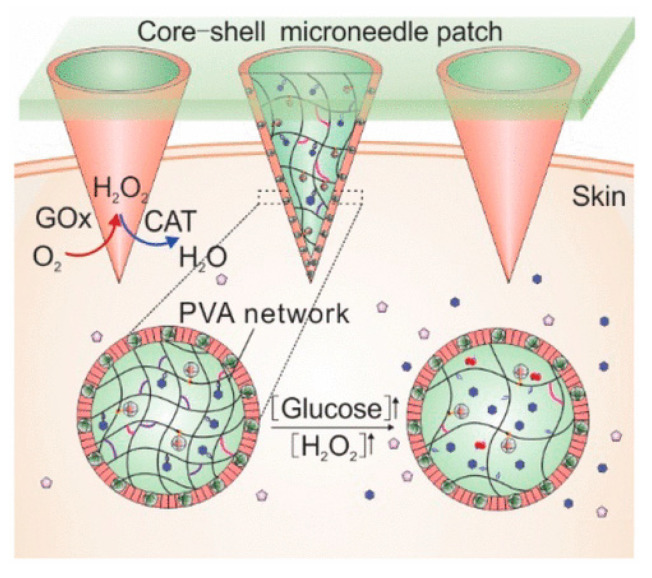
Mechanism of glucose-stimulated insulin release via glucose oxidase in a microneedle array patch. Adapted with permission from [65]; Published by ACS Nano, 2018.

Wang et al. created a degradable cross-linked gel, core–shell microneedle array patch for smart insulin administration coupled with a quick response and great biocompatibility. Figure 7 depicts a graphical representation of the stimuli responsive system. The hydrogel system partially dissolves and then releases insulin when stimulated by hydrogen peroxide (H_2_O_2_). H_2_O_2_ is produced during the oxidation of glucose by a glucose-specific enzyme covalently bonded inside the gel. Moreover, the microneedles, which are H_2_O_2_-responsive, are covered with a thin film carrying an H_2_O_2_-scavenging enzyme, imitating the complimentary role of peroxisome enzymes in protecting normal tissues from injury via oxidative stress. The researchers demonstrated in a T1D model that the formulated smart insulin patch with a protective shell and bioresponsive core could maintain normoglycemia with improved biocompatibility. The core–shell gelated MN array patch is suitable for glucose-stimuli smart insulin delivery. The MN gels were made utilizing a “solution-gelation” process that involved the layering of diluted solution [65].

**Figure 8:**
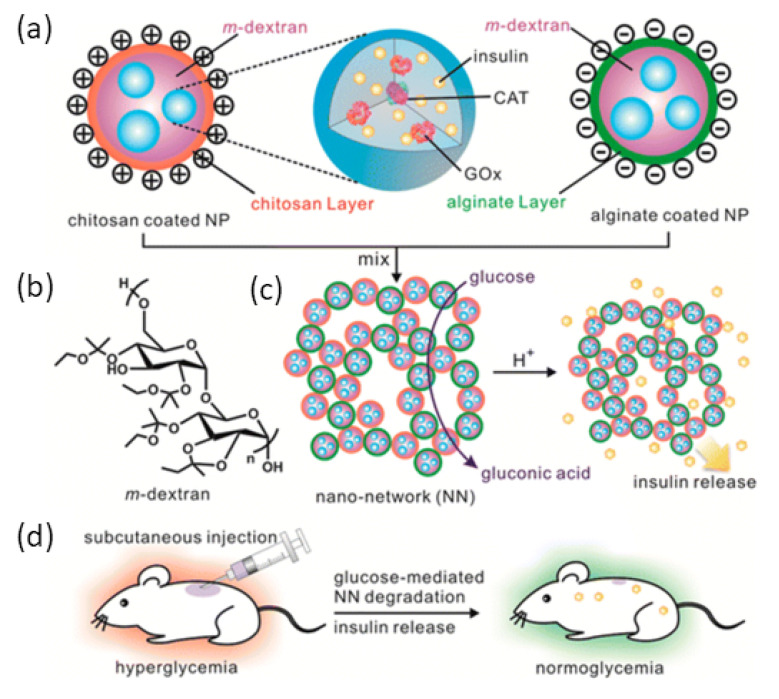
(**a**) Polymeric NPs functionalized with glucose-specific enzymes, CAT and GOx, and insulin. (**b**) m-dextran coated with CHC and alginate, respectively. (**c**) Depiction of the nano-network and stimulated insulin release via GOx conversion to gluconic acid. (**d**) SC insulin delivery for the treatment of DM in STZ-induced T1D mice. Adapted with permission from [66]; Published by ACS Nano, 2013.

Figure 8 depicts the injectable nanonetwork of Gu et al. comprised of dextran nanoparticles. The acidic environment damaged the nanonetworks, which then were encapsulated with insulin, catalase, and GOx. Hyperglycemia causes the insulin to dissociate and release at the same time. To create a porous nanonetwork, alginate and CHC were utilized to coat acid-sensitive dextran to produce positively and negatively charged particles. In the presence of high BG levels, GOx converts glucose to gluconic acid, causing a pH shift and the release of insulin. This nanotech method was proven in vitro, demonstrating that a single injection maintained homeostatic glucose management (<200 mg/dL) for over a week in T1D mice when delivered subcutaneously within a degradable nano-network [66].

An interesting study carried out in North Carolina, USA demonstrated the use of ketal-modified dextran nanoparticles integrated within a hyaluronic acid microgel. The nanoparticles were glucose responsive by encapsulating GOx and insulin. The results from this study demonstrated an insulin release corresponding to changes in glucose levels in vitro. Animal studies were also carried out in STZ-induced T1D mice. Data showed a negligible toxicity and that a single subcutaneous injection maintained the normoglycemic state for over 7 days [67].

Li et al. created a pH-sensitive peptide hydrogel to transport insulin, Gox, and catalase. Under physiological conditions, the peptide was able to self-assemble into a hydrogel. As there was an increase in blood glucose and the pH of the system decreased, insulin was released via deconstruction of the peptide hydrogel. On the contrary, as blood glucose levels fell and pH increased, the peptide hydrogels were able to reassemble, stopping the release of insulin while GOx regulated the local pH. At a low enough pH, the electrostatic repulsion between the charged lysine/ornithine side groups resulted in the unfolding of hairpins. As these peptide hydrogels effectively demonstrated control of blood glucose, they were considered promising biomaterials for diabetes therapy. Experiments in vivo demonstrated the hydrogel was injectable, biocompatible, and efficient in regulating blood glucose over time [68].

### 5.2. Glucose-Responsive Stimuli Release Systems

**Figure 9:**
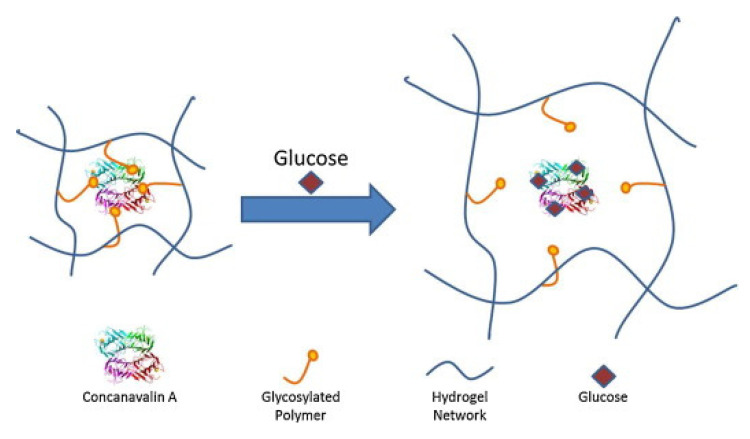
Illustration of hydrogel network swelling for insulin release via ConA’s affinity for glucose. Adapted with permission from [58]; Published by Elsevier, 2015.

Concanavalin A (ConA) is derived from the jack bean plant and is a non-sugar protein. ConA has a highly reversible affinity for unmodified hydroxyl groups at carbons 3 to 4 and 6 of pyranose ring-containing glycopolymers, monosaccharides and polysaccharides, such -D-glucose [69]. The four molecules of ConA can form a tetramer complex with unaltered pyranose rings under physiological pH. With a higher affinity for glucose than glycosylated polysaccharides and glycopolymers, ConA can be used to make glucose-sensitive hydrogels, as shown in Figure 9 [59,70]. ConA binds to the hydrogel’s glycosylated moieties, resulting in a highly complexed matrix with small pore diameters that trap insulin within the hydrogel. Glucose has a higher affinity for ConA than glycosylated moieties; therefore, glucose is able to displace the glycosylated polymer, resulting in the hydrogel either swelling and releasing insulin via diffusion or exhibiting a gel–sol transition and releasing insulin via matrix liberation [58].

ConA, on the other hand, may leak from a low viscosity sol, resulting in decreased glucose sensitivity as well as adverse cytotoxicity and immunogenicity. To circumvent these limitations, ConA has been used in combination with polymers to synthesize chemically cross-linked hydrogels. For example, Taylor et al. created a closed-loop system via a cross-linked ConA–dextran hydrogel for the administration of insulin. The hydrogel demonstrated real-time glucose sensitivity within an implantable artificial pancreas in a diabetic domestic pig [71].

**Figure 10:**
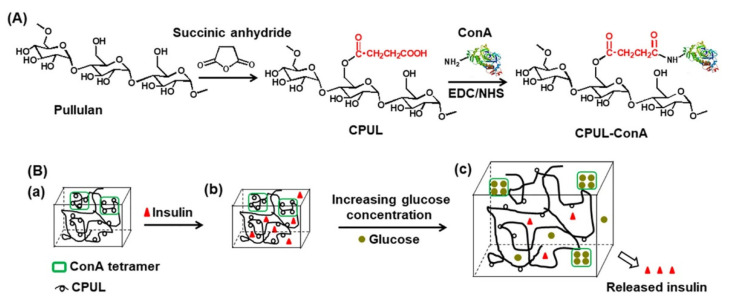
(**A**) Preparation of the CPUL-ConA hydrogel via EDC/NHS crosslinking. (**B**) Mechanism of controlled release of insulin from the CPUL-ConA hydrogel. (**a**) The ConA tetramer with the carboxylated Pullulan hydrogel. (**b**) Insulin drug added to the ConA-CPUL hydrogel. (**c**) Increasing glucose concentration in hydrogel environment demonstrating glucose responsiveness. Adapted with permission from [72]; Published by Elsevier, 2019.

In a pullulan-based glucose responsive hydrogel with a linear glucan structure, the possibility of intelligently regulated insulin release in proportion to fluctuations in blood glucose was examined. Figure 10A depicts the formation of a modified pullulan having –COOH groups (CPUL). A hydrogel comprising of covalently altered carboxylated pullulan and ConA (ConA-CPUL) was produced to limit ConA leakage by activating it with 1-ethyl-3-(3-dimethylaminopropyl) carbodiimide/*N*-hydroxysuccinimide (EDC/NHS). Figure 10B shows the ConA-CPUL (insulin/ConA-CPUL) insulin-loaded hydrogel and glucose responsiveness via smart regulated insulin release in vitro. Studies carried out in vitro to determine drug release profiles revealed that the smart controlled release of insulin from the drug-loaded hydrogels was possible due to ConA-glucose binding. The viscoelasticity and shear thinning characteristics of covalently altered hydrogels were observed in the insulin/CPUL-ConA hydrogel. SEM images revealed a cross-linked network topologies with homogeneous porosity and insulin particles. As a result, this mechanism can provide glucose-responsive insulin release [72].

Yin et al. used reversed-phase emulsion crosslinking to create glucose-sensitive microgels to determine if genipin-crosslinked ConA/GEA-CHC microgels may be employed as a glucose-sensitive DDS. In this work, the ConA polymer ligand was produced as a glucosyloxyethyl acrylated CHC derivative (GEA-CHC) that could be quickly crosslinked with ConA by genepin, avoiding ConA alteration during the immobilization process. In vitro analysis revealed that the microgels were non-toxic and that the released insulin remained active without altering its tertiary structure. ConA leakage was also limited as a result of genepin crosslinking [69].

### 5.3. Temperature/Thermo-Responsive Stimuli Release of Insulin

The structure of thermo-responsive hydrogels has a hydrophobic–hydrophilic balance. The collapse or relaxation of the hydrogel chain occurs when the temperature changes near the critical solution temperature (CST). This is in reaction to changes in the hydrophilic and hydrophobic interactions between hydrogel polymer chains and water molecules. The temperature at which the polymer solution divides from one phase to two phases is referred to as the CST [73]. Temperature-sensitive hydrogels have the capacity to swell/deswell in response to environmental temperature variations [62]. This characteristic is heavily influenced by the upper (UCST) or lower critical solution temperature (LCST). Dehydration occurs in the UCST when the ambient temperature is below the LCST (i.e., it is soluble at higher temperatures), but deswelling occurs when the ambient temperature is above the LCST (i.e., it is soluble at lower temperature). Since the polymer solution may undergo thermally induced self-assembly (post injecting) to form a hydrogel at 37 °C, polymers with an LCST/phase transition between ambient temperature and 37 °C have been used to synthesize injectable in situ hydrogels [74].

Lee et al. used pentablock copolymers to produce a hybrid thermo-responsive platform for insulin delivery. The copolymers used were oligomer serin-b-poly (lactide)-b-poly (ethylene glycol)-b-poly (lactide)-b-oligomer serine (OS-PLA-PEG-PLA-OS). They developed nanospheres were uniformly dispersed throughout the matrix, reinforcing the mechanical characteristics of the hydrogel depot and prolonging its degradation period under physiological circumstances. To assess the in vitro insulin-release profile, the hydrogel–nanosphere system was subcutaneously injected into diabetic BALB/c rodents. The results demonstrated that the concentration of insulin in the blood remained steady. The insulin’s bio-property was preserved and demonstrated blood glucose lowering for longer than 60 h post administration into a streptozotocin (STZ)-induced diabetic rodent model. This indicates that the injectable pH-temperature sensitive hydrogel containing CHC-insulin electrosprayed nanosphere composites has promising potential applications for T1D therapy [75].

### 5.4. Metal-Conjugated Platforms for Potential Insulin Delivery

Hydrogels that are sensitive to electric and magnetic fields modify their characteristics stimuli responsively to minor variations in electric current or external fields. These systems have been studied as swellable, shrinkable, and bendable hydrogels. These hydrogels have been extensively researched as polyelectrolyte hydrogels (systems that contain a high concentration of ionizable) [76]. Polyvinyl alcohol, acrylic acid/vinyl sulfonic acid, and sulfonated polystyrene are some of the synthetic polymers utilized to produce electroactive hydrogels. Natural polymers are also often used in the fabrication of electro- and magnetically sensitive devices. Hyaluronic acid, alginate, and CHC, for example, have been used in combination with synthetic polymers to create electroactive hydrogels [35,76].

## 6. The Use of 3D Bio-Printing to Engineer an Artificial Pancreas and Other 21st Century Techniques

The idealized solution to the diabetes mellitus pandemic would be islet and pancreatic transplantation for endogenous insulin release. However, the transplantations are plagued with problems. Diabetic patients have to undergo surgery, which is only possible if organs are readily available, and thereafter, the individual will be on chronic immunosuppression, which is not ideal as T1D often manifests in children [77]. Thus, the introduction of 3D printing in the biological field opened the doors to the regeneration and replacement of biological tissue and organs (bioimplants) for the development of the artificial pancreas [78]. This is important, as the printed scaffold can be placed at the targeted site where tissue regeneration/organ replacement can take place. Both natural and synthetic polymers as well as bioinks, which are living cells contained within a cellular matric, are used in 3D bioprinting [78].

As the treatment of T1D results in poor patient compliance and unwanted side effects, in 2019, researchers (Kim et al., 2019) strived to develop a system that could potentially regenerate pancreatic β-cell function. They synthesized a pancreatic tissue-derived extracellular matrix bioink, and using both child and adult islet cells, they were able to validate that the bioink fulfilled the criterion for tissue engineering and could work as a 3D construct [79].

Three-dimensional (3D) printing may take place via inkjet, laser printing, stereolithography, or via extrusion [77]. The extrusion method of 3D printing was carried out by Duin et al. to formulate a methylcellulose/alginate hydrogel loaded with pancreatic islets. The data showed that the islets were still able to produce insulin and glucagon and demonstrated slight glucose responsiveness. The researchers loaded the islet tissue within a hydrogel to protect it from physiological degradation within the body; this was confirmed when the morphology and α and β cells within the islets were maintained [80].

However, as with emerging technology, there are challenges that need to be addressed. The time taken to construct the material, the management of cell viability, and crucially the biocompatible materials for synthesis, which can be very costly, are some of the limitations to 3D bioprinting [78].

## 7. Future Perspectives

Emerging insulin administration techniques have the potential to dramatically improve patient compliance to achieve glycemic control and improved quality of life and delay/reduce risk of complications and death. Smart insulin delivery systems that respond to external stimuli and adapt to individual physiological circumstances to provide regulated insulin release are at the forefront of T1D treatment research. The design, synthesis, characteristics, and use of smart hydrogels in biomedical engineering have undergone a paradigm shift in the last decade [35]. As extensively outlined by Kondiah et al., various stimuli systems have been evaluated for various controlled release kinetics as well as rapid release systems, encapsulating proteins of various nature, as well as tissue engineered delivery systems for optimal therapeutic efficacy for future applications [81]. Islet-loaded hydrogels are a promising area for the possible regeneration of T1D β-cells. As a result, regenerative medicine and tissue engineering are inextricably linked with the controlled delivery of insulin [8]. Islet transplantation has been performed in over 1500 patients. However, many hurdles remain such as immunotherapy, a lack of donors, and importantly, researchers are yet not able to reproduce the microenvironment conditions of the islet cells [82]. Although considerable progress has been made, the practical application of hydrogels is limited by the issues of complex production process and high cost. Therefore, an ideal prolonged delivery system for insulin overcoming the mentioned issues is still required [4]. However, with a greater understanding of local environmental circumstances, cellular and metabolic processes in the healthy and pathologic states, and the chemicals available for the creation of injectable hydrogels, the administration of diabetic treatment can easily be transferred from research to clinical reality. As a result, the utilization of injectable systems for therapeutic administration is seen to have considerable promise [6].

## 8. Conclusions

To adapt to physiological changes, stimuli-responsive hydrogels have been synthesized. When specialized, they become particularly appealing for various biological applications due to their unique properties of controlled swelling, biocompatibility, biodegradability, and preserving fluid within their networked structures. Injectable hydrogels can enhance the controlled distribution and prolonged release of insulin in both systemic and localized settings. Hydrogels can be used for patient-centric advances to be made in insulin delivery by offering spatio-temporal control over the administered insulin. Modified hydrogels as smart systems can satisfy a key requirement in controlled injectable insulin delivery based on their chemical composition and method of synthesis. The fundamental paradox in the use of mechanical characteristics of hydrogels for controlled insulin delivery is that increasing the polymer concentration increases the mechanical strength at the expense of poor biocompatibility and biodegradability. A therapeutically appropriate design for oral insulin has not yet been proven successful; however, attempts are still being made in this area. The next generation of smart insulin delivery systems should impress rapid response, simplicity of preparation and administration, and high biocompatibility. Thus, injectable ‘smart’ hydrogels remain the forerunner for the development of advanced patient-centric therapy for T1D. Further in vivo testing of the response to varied glucose levels is required before clinical translation of the various technologies.

## Figures and Tables

**Table 1:** Describing different labile bonds present in hydrogels, including their functional groups and chemical structures, for the release of therapeutic agents. Adapted with permission from [20]; Published by MDPI, 2019.

Linker Type	Chemical Structure	Degradation Conditions	References
Acetalic linker	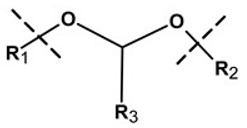	Hydrolysis in acidic medium, pH = 5	[22]
Ketal linker	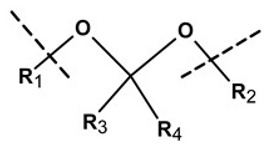	Hydrolysis in acidic medium, pH = 5.5	[23]
Ester linker	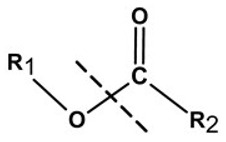	Hydrolysis below physiological pH	[24]
Vinyl ether linker	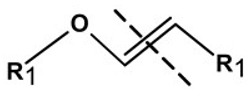	Hydrolysis in acidic medium, pH < 5	[25]
Linker based on ortho-nitrobenzyl ester	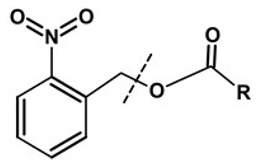	Hydrolysis under the influence of UV 315–390 nm	[26]
Linker based on disulfide or diselenide bridges	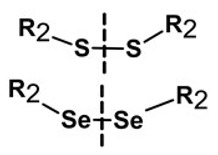	Hydrolysis in the presence of GSH carboxyethylphosphine tris (TCEP), and dithiothreitol (DTT)	[27,28,29]
Phosphoester linker	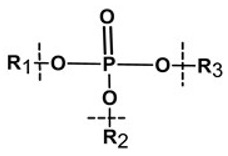	Hydrolysis in the presence of phosphatase or phospholipase enzyme	[30]

**Table 2:** Micro- and nano-hydrogel systems for insulin delivery. Including polymers utilized, sustained release time, and whether they have been tested in cell or animal models.

System Employed	Polymers Utilized	Insulin Encapsulation Efficiency (EE)/Loading Capacity (LC) (%)	Insulin Release Time	In Vitro or In Vivo Studies Carried Out	References
Nanoparticles within microgel	-Alginate -Acetylated dextran	39% -EE 6.5% -LC	22 days (2 doses) in vivo	In vitro and in vivo studies carried out	[45]
Microgel encapsulated microspheres	-CHC (Chitosan)	62.96 ± 0.68% -EE	7 days in vitro	In vitro studies	[46]
Ultrasound triggered nanocapsules within microgel	-PLGA -CHC	71.3 ± 1.8% -EE 11.9 ± 0.6% -LC	10 days in vitro 7 days in vivo	In vitro and in vivo studies carried out	[47]
Self-assembled nanoparticles in gel (nanogel)	-Carboxymethyl-hexanoyl CHC	Insulin loaded 5 mg/mL	10 days in vivo	In vitro and in vivo studies carried out	[48]
Monodispersed nanogels	-Poly(*N*-isopropylacrylamide) -Dextran -Poly(3-acrylamidophenylboronic acid)	80.6% -EE 16.2% -LC	2 h in vivo	In vitro and in vivo studies carried out	[49]
Double-layered nanogel	-Glycol CHC -Sodium alginate poly (L-glutmate-co-*N*-3-L-glutamylphenylboronic acid)	71 ± 3.5% -LC	3 h in vivo	In vitro and in vivo studies carried out.	[50]

## Data Availability

Not applicable.
